# Network Pharmacology Reveals the Molecular Mechanism of Cuyuxunxi Prescription in Promoting Wound Healing in Patients with Anal Fistula

**DOI:** 10.1155/2019/3865121

**Published:** 2019-09-17

**Authors:** Yin Qu, Zhijun Zhang, Yafeng Lu, De Zheng, Yang Wei

**Affiliations:** Department of Anorectal Surgery, Shuguang Hospital, Shanghai University of Traditional Chinese Medicine, Shanghai 201210, China

## Abstract

**Background:**

The healing process of the surgical wound of anal fistulotomy is much slower because of the presence of stool within the wound. Cuyuxunxi (CYXX) prescription is a Chinese herbal fumigant that is being used to wash surgical wound after anal fistulotomy. This study aimed at investigating the molecular mechanism of CYXX prescription using a network pharmacology-based strategy.

**Materials and Methods:**

The active compounds in each herbal medicine were retrieved from the traditional Chinese medicine systems pharmacology (TCMSP) database and in Traditional Chinese Medicine Integrated Database (TCMID) analysis platform based on the criteria of oral bioavailability ≥40% and drug-likeness ≥0.2. The disease-related target genes were extracted from the Comparative Toxicogenomics Database. Protein-protein interaction network was built for the overlapped genes as well as functional enrichment analysis. Finally, an ingredient-target genes-pathway network was built by integrating all information.

**Results:**

A total of 375 chemical ingredients of the 5 main herbal medicines in CYXX prescription were retrieved from TCMSP database and TCMID. Among the 375 chemical ingredients, 59 were active compounds. Besides, 325 target genes for 16 active compounds in 3 herbal medicines were obtained. Functional enrichment analysis revealed that these overlapped genes were significantly related with immune response, biosynthesis of antibiotics, and complement and coagulation cascades. A comprehensive network which contains 133 nodes (8 disease nodes, 3 drug nodes, 8 ingredients, 103 target gene nodes, 7 GO nodes, and 4 pathway nodes) was built.

**Conclusion:**

The network built in this study might aid in understanding the action mechanism of CYXX prescription at molecular level to pathway level.

## 1. Introduction

Anal fistula is characterized by chronic abnormal communication between the epithelialized surface of the anal canal and the perianal skin [1]. The incidence and epidemiology of anal fistula were estimated as 8.6 per 100,000 population for nonspecific fistula, with 12.3 per 100,000 for males and 5.6 for females [2]. The incidence of anal fistula is increasing annually in the European Union [3], which significantly affects patients' quality of life. Treatment of anal fistula is a challenge for physicians for thousand years. The most frequently used therapy for treating anal fistula is surgery, such as simple fistulotomy [4]. However, it is associated with a significant risk of recurrence and complications such as fecal incontinence [5, 6]. Besides, the surgical wound infection is hard to healing and easy to occur because of its special location. Therefore, strategies for reducing the incidence of complications are urgently needed for physicians.

Traditional Chinese medicine (TCM) plays an important role in maintaining health for Asian people. TCM has attracted the most attention for western countries in these years because of its reliable therapeutic efficacy and fewer side effects [7, 8]. In recent years, fumigation-washing therapy on the surgical wound using Chinese herbal fumigant has been widely used for postoperative treatment and achieved satisfied effect. Cuyuxunxi (CYXX) prescription is a Chinese herbal fumigant composed of *Reynoutria japonica* Houtt. (30 g), *Taraxacum mongolicum* Hand.-Mazz. (30 g), *Rhus chinensis* Mill. (15 g), *Sophora flavescens* Aiton (9 g), and *Angelica sinensis* (Oliv.) Diels (9 g). Our clinical studies have proved that long-time treatment with a low concentration of CYXX prescription can relieve pain, pruritus, turgescence, and effusion after anal fistulotomy and promote surgical wound healing [9]. However, the underlying mechanisms have seldom investigated.

The molecular mechanism of TCM, especially TCM formulae, is difficult to investigate previously because of the complex chemical composition and complex interactions among multiple components. Network pharmacology is a new method based on the theory of network biology. It has made a significant contribution to investigate the molecular mechanisms of TCM by combining pharmacokinetic evaluation and bioinformatics. In the past decades, tens of studies have proven the feasibility of network pharmacology in investigating the molecular mechanism of TCM [10–13]. In this study, we aimed at investigating the mechanisms of CYXX prescription in promoting surgical wound healing using a comprehensive network pharmacology-based approach.

## 2. Materials and Methods

### 2.1. Search for Chemical Ingredients

The traditional Chinese medicine systems pharmacology database and analysis platform (TCMCP) (http://sm.nwsuaf.edu.cn/lsp/tcmsp.php) contains large number of herbal entries, drug-target networks, and drug-disease networks, which help researchers in revealing the mechanisms of action of Chinese herbs [14]. The information of each herbal medicine, including the ingredients, molecule name, molecular weight (MW), water partition coefficient (*A*  log  *P*), number of hydrogen bond donors and receptors (Hdon/Hacc), human oral bioavailability (OB), half-life (HL), Caco-2 permeability (Caco-2), blood-brain barrier (BBB), and drug-likeness (DL), was obtained. For the herb whose information was not obtained from TCMCP database, we further searched its information in Traditional Chinese Medicine Integrated Database (TCMID) (http://www.megabionet.org/tcmid/), which is a comprehensive database aiming at TCM's modernization and standardization [15].

### 2.2. Screening for Active Compounds

The active compounds were screened out on the basis of the ADME processes. The parameters of ADME include OB, DL, and HL. In this study, the compounds with OB ≥ 40% and DL ≥ 0.2 were regarded as active compounds.

### 2.3. Identification of Target Proteins of Active Compounds

DrugBank database (http://www.drugbank.ca) is a unique bioinformatics and cheminformatics resource that combines detailed drug data with comprehensive drug-target information [16, 17]. We searched the direct target proteins of each chemical ingredient in CYXX prescription from the DrugBank database. The full name of the target proteins was converted to gene symbol based on the UniProt ID in the UniProt database (https://www.uniprot.org/) for further analysis.

### 2.4. Screening of Disease-Related Genes

Comparative Toxicogenomics Database (CTD, http://ctdbase.org/) is a cross-species resource for building chemical-gene interaction networks [18, 19]. This database facilitates identification and understanding of chemical-gene-disease associations [20]. We searched the disease-related genes in CTD using the keywords of “acute pain,” “edema,” “angioedema,” “surgical wound dehiscence,” “surgical wound infection,” “wound infection,” “skin ulcer,” “pyoderma gangrenosum,” “cutaneous fistula,” “fissure in Ano,” “fasciitis,” and “Necrotizing.” The intersection between the disease-related genes obtained from CTD and the target genes of active compounds was reserved for further analysis.

### 2.5. Functional Enrichment Analysis

The Database for Annotation, Visualization, and Integrated Discovery (DAVID, https://david.ncifcrf.gov/) v6.8 provides a comprehensive set of functional annotation tools for investigators to understand biological meaning behind large list of genes. In order to investigate the functions of the target genes, we performed gene ontology (GO) biological process (BP) enrichment analysis and Kyoto Encyclopedia of Genes and Genomes (KEGG) pathway enrichment analysis using DAVID. *P* value <0.05 was regarded as the significance cutoff in this study.

### 2.6. Protein-Protein Interaction (PPI) Network Construction

STRING database contains all publicly available sources of PPI information [21]. The interactions of the overlapped target genes were predicted using the STRING database based on the cutoff criterion of required confidence >0.4. The PPIs satisfied this criterion were downloaded and submitted to Cytoscape software [22] (version 3.6.1, Boston, MA, USA) for visualizing the PPI network.

### 2.7. Construction of Ingredient-Target Genes-Pathway Network

The predicting target genes of each ingredient of CYXX prescription, the disease-related genes, and the GO-BP or KEGG pathway involved were connected to build an integrated network to illustrate the pharmacological mechanisms of CYXX prescription. The graphical interaction of this network was visualized by Cytoscape software.

## 3. Results

### 3.1. Identification of Target Genes of CYXX Prescription

**Figure 1:**
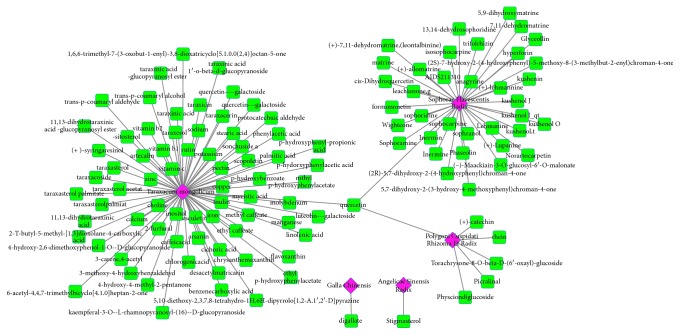
The herb-active compounds network. A compound node and an herb are linked if the compound is in the herb.

The CYXX prescription consists of 5 main herbal medicines, Sophorae Flavescentis Radix, Polygoni Cuspidati Rhizoma Radix, Angelicae Sinensis Radix, Galla Chinensis, and *Taraxacum mongolicum*. We searched these keywords in TCMSP database. A total of 113, 62, 125, 3, and 0 chemical ingredients of the five herbal medicines in CYXX prescription were retrieved from TCMSP database. Since there was no result for *Taraxacum mongolicum* in TCMSP, we further searched its chemical ingredients in TCMID using the keyword of “PU GONG YING” and obtained 72 chemical ingredients. Based on the criteria of OB ≥ 40% and DL ≥ 0.2, a total of 37, 6, 1, 1, and 14 active compounds were identified for these 5 herbal medicines finally (Table 1). A herb-active compounds network was constructed by using Cytoscape (Figure 1).

The direct target proteins of each chemical ingredient in CYXX prescription were searched from the DrugBank database, and 345 target genes for 16 active compounds in 3 herbal medicines were obtained (Supplementary [Supplementary-material supplementary-material-1]).

### 3.2. Screening of Disease-Related Genes

Since the CYXX prediction is used for anti-infection and promoting wound healing after anal fistula surgery, we searched disease related with anti-infection and wound healing in CTD. The overlapped genes between the disease-related genes obtained from CTD and the target genes of active compounds were identified. Finally, we obtained a total of 313 overlapped genes between 8 disease-related genesets and the target genes of active compounds (Table 2).

### 3.3. Functional Annotation Analysis of the Overlapped Genes

GO-BP and KEGG pathway enrichment analyses were further performed for the overlapped genes. As shown in Table 3, these genes were significantly related with KEGG pathways of “complement and coagulation cascades” (*P*=1.90*E* − 28), “biosynthesis of antibiotics” (1.70*E*−04), “HIF-1 signaling pathway” (*P*=5.15*E* − 04), and “antigen processing and presentation” (*P*=0.02474). Besides, the GO-BP terms of “platelet degranulation” (*P*=5.95*E* − 24), “complement activation, classical pathway” (*P*=2.80*E* − 19), “regulation of complement activation” (*P*=1.91*E* − 15), “complement activation” (*P*=4.28*E* − 12), and “innate immune response” (*P*=1.94*E* − 11) were significantly enriched.

### 3.4. Construction of Ingredient-Target Genes-Pathway Network

**Figure 2:**
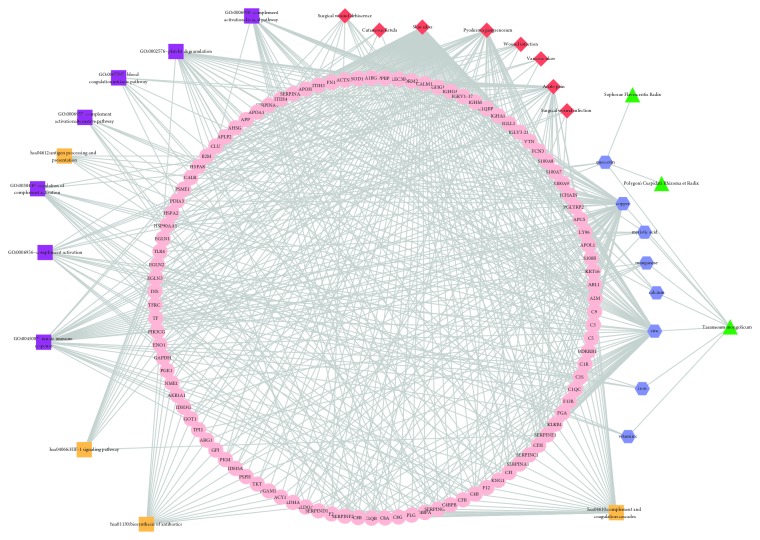
The ingredient-target genes-pathway network.

A comprehensive network contains 133 nodes (8 disease nodes, 3 drug nodes, 8 ingredients, 103 target gene nodes, 7 GO nodes, and 4 pathway nodes) was built by integrating all information (Figure 2). For all the 8 disease nodes, the degree of “skin ulcer” was the highest (degree = 103) and the next one was “pyoderma gangrenosum” (degree = 30). For the ingredients, zinc regulated the greatest number of target genes (degree = 60) and the subsequent one was copper (degree = 58).

**Figure 3:**
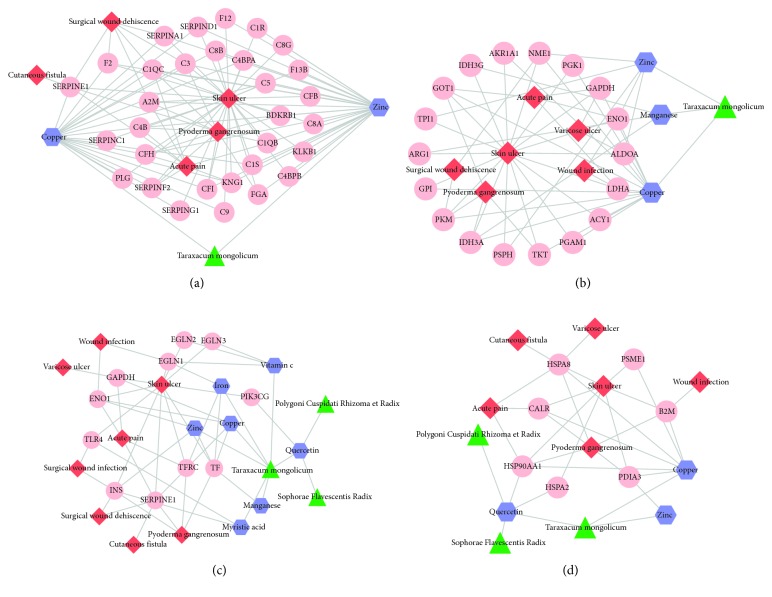
Subnetwork analysis.

We further divided the comprehensive network into several subnetworks based on our interested pathways: hsa04610: complement and coagulation cascades, hsa01130: biosynthesis of antibiotics, hsa04066: HIF-1 signaling pathway, and hsa04612: antigen processing and presentation (Figure 3). From Figure 3(a), we could find that genes in the pathway of complement and coagulation cascades were closely related with the diseases of skin ulcer, surgical wound dehiscence, cutaneous fistula, acute pain, and pyoderma gangrenosum as well as the small molecules of zinc and copper. Both of these two small molecules were derived from *Taraxacum mongolicum*. From Figure 3(b), the small molecules of zinc, copper, and manganese derived from *Taraxacum mongolicum* were closely related with genes in the pathway of biosynthesis of antibiotics, which is a pathway that being disturbed in diseases of acute pain, skin ulcer, surgical wound dehiscence, pyoderma gangrenosum, and wound infection. From Figure 3(c), quercetin derived from Polygoni Cuspidati Rhizoma et Radix, *Taraxacum mongolicum*, and Sophorae Flavescentis Radix could regulate the expression of PI3KCG, which was dysregulated in skin ulcer. Besides, the small molecules iron, copper, zinc, vitamin C, manganese, and myristic acid from *Taraxacum mongolicum* could regulate the expression of SERPINE1, TFRC, EGLN1, and TLR4 in HIF-1 signaling pathway. These genes were frequently dysregulated in several diseases, including wound infection, skin ulcer, acute pain, surgical wound infection, cutaneous fistula, and pyoderma gangrenosum. In Figure 3(d), we could find that quercetin could regulate the expression of HSP90AA1 and HSPA2 in the pathway of antigen processing and presentation, which were dysregulated in diseases of skin ulcer and acute pain.

## 4. Discussion

TCM has been widely used in China for thousands of years. Though TCM has reliable therapeutic efficacy and fewer side effects, it is hard to be accepted by western countries because of its complex chemical composition and uncertain theory basis. The emergence of network pharmacology owning to the development of systems biology, bioinformatics, and polypharmacology has advanced the investigation of TCM into a new period [23–25]. By shifting from “one-target, one-drug” analysis to “network-targeted, multicomponent” analysis, network pharmacology is a powerful way for the molecular mechanism of TCM formula [8, 26].

In this study, we applied a network pharmacology-based strategy to investigate the molecular mechanism of CYXX prescription in relieving pain and promoting surgical wound healing after anal fistulotomy. A total of 375 chemical ingredients of the 5 main herbal medicines in CYXX prescription were retrieved from TCMSP database and TCMID. Among the 375 chemical ingredients, 59 active compounds that could overcome the barriers posed by absorption, distribution, metabolism, and excretion (ADME) processes were identified [27]. The recommended thresholds of OB and DL are 30% and 0.18 in TCMSP [14], respectively. In this study, we set the criteria of OB and DL as 40% and 0.20, which were higher than the recommended threshold. These criteria were set based on our experience aimed at obtaining more reliable results. These data additionally indicate the potential of CYXX prescription as a promising therapy for surgical wound healing.

By searching the direct target proteins from the DrugBank database, 345 target genes for 16 active compounds in 3 herbal medicines were obtained. Meanwhile, target genes of 8 wound healing or anti-infection-related diseases were searched from CTD and the overlapped target genes between these two datasets were selected as candidates for further analysis. Compared to the surgical wound on other position, the healing process of the surgical wound of anal fistulotomy is much slower because of the presence of stool within the wound [5]. CYXX prescription is a TCM formula based on several years of clinical experiences. This prescription has been used for postoperative treatment for patients underwent anal fistulotomy. Results suggested that this prescription could relieve pain, pruritus, turgescence, and effusion and promote surgical wound healing [9]. Therefore, we speculated the molecular mechanism of CYXX prescription might be related with anti-infection. Therefore, diseases related with “pain,” “turgescence,” “edema” were searched from CTD including “acute pain,” “edema,” “angioedema,” “surgical wound dehiscence,” “surgical wound infection,” “wound infection,” “skin ulcer,” “pyoderma gangrenosum,” “cutaneous fistula,” “fissure in Ano,” “fasciitis,” and “Necrotizing.” Finally, 313 target genes of 8 diseases were obtained.

GO-BP and KEGG pathway enrichment analysis revealed that these overlapped genes were significantly related with immune response, biosynthesis of antibiotics, and complement and coagulation cascades. In the comprehensive ingredient-target genes-pathway network, we identified several active compounds in CYXX prescription that could act on these pathways. For example, genes in the pathway of complement and coagulation cascades were closely related with the diseases of skin ulcer, surgical wound dehiscence, cutaneous fistula, acute pain, and pyoderma gangrenosum as well as the small molecules of zinc and copper. Both of these two small molecules were derived from *Taraxacum mongolicum*. The complement system, as part of innate immunity, is activated immediately after trauma [28]. The complement system plays a central role in innate immunity, and the coagulation system is the main column in hemostasis which undergoes massive activation early after injury [29]. The humoral serine proteases in this pathway play central roles during the events of an inflammatory response [30]. In our subnetwork, serine proteases in coagulation and complement system, including SERPINE1, SERPINA1, SERPIND1, SERPINC1, SERPINF2, and SERPING1, are target genes of copper and zinc, which were derived from *Taraxacum mongolicum*. Therefore, we hypothesized that *Taraxacum mongolicum* in CYXX prescription contributes to the role of relieving pain and promoting wound healing. These results further confirmed the reliability of our analysis and the role of CYXX prescription in clinic.

Despite the significant new findings of this study, some limitations still exist. This study is a primary study for investigating the molecular mechanism of CYXX prescription based on network pharmacology. Though this method has been proven effective by most studies, experimental validation for the network is still warranted for our study.

In conclusion, our results provide the theory basis for CYXX prescription in reducing complications and promoting wound healing after anal fistulotomy. The network built in this study might shed some new lights on understanding the pathogenesis of postoperative complications and aid in understanding the action mechanism of CYXX prescription at molecular level to pathway level. However, further experiments are warranted to validate the relationships of the network in this study.

## Figures and Tables

**Table 1:** The active compounds in the five main herbal medicines of Cuyuxunxi prescription.

Herbs	Mol ID	Molecule name	MW	*A* log *P*	OB (%)	Caco‐2	BBB	DL
Sophorae Flavescentis Radix	MOL001040	(2R)-5,7-Dihydroxy-2-(4-hydroxyphenyl)chroman-4-one	272.27	2.3	42.36	0.38	−0.48	0.21
	MOL001484	Inermine	284.28	2.44	75.18	0.89	0.4	0.54
	MOL003627	Sophocarpine	246.39	1.39	64.26	0.99	1	0.25
	MOL003648	Inermin	284.28	2.44	65.83	0.91	0.36	0.54
	MOL003673	Wighteone	338.38	3.92	42.8	0.64	−0.16	0.36
	MOL003676	Sophoramine	244.37	1.15	42.16	1.43	1.53	0.25
	MOL003680	Sophoridine	248.41	1.42	60.07	1.13	1.14	0.25
	MOL000392	Formononetin	268.28	2.58	69.67	0.78	0.02	0.21
	MOL004580	*cis*-Dihydroquercetin	304.27	1.49	66.44	−0.34	−1.11	0.27
	MOL005100	5,7-Dihydroxy-2-(3-hydroxy-4-methoxyphenyl)chroman-4-one	302.3	2.28	47.74	0.28	−0.3	0.27
	MOL005944	Matrine	248.41	1.42	63.77	1.39	1.52	0.25
	MOL006562	(+)-7,11-Dehydromatrine, (leontalbinine)	246.39	1.42	62.08	1.06	1.12	0.25
	MOL006564	(+)-Allomatrine	248.41	1.42	58.87	1.08	1.13	0.25
	MOL006565	AIDS211310	248.41	1.42	68.68	1.15	1.38	0.25
	MOL006566	(+)-Lehmannine	246.39	1.11	58.34	1.21	1.36	0.25
	MOL006568	Isosophocarpine	246.39	1.39	61.57	1.39	1.45	0.25
	MOL006571	Anagyrine	244.37	1.15	62.01	1.16	1.13	0.24
	MOL006573	13,14-Dehydrosophoridine	246.39	1.39	65.34	1.06	1.11	0.25
	MOL006582	5*α*,9*α*-Dihydroxymatrine	280.41	−0.3	40.93	0.04	−0.43	0.32
	MOL006583	7,11-Dehydromatrine	246.39	1.42	44.43	1.11	1.13	0.25
	MOL006596	Glyceollin	338.38	2.85	97.27	0.53	−0.19	0.76
	MOL003347	Hyperforin	536.87	8.62	44.03	0.87	0.4	0.6
	MOL006604	(2S)-7-Hydroxy-2-(4-hydroxyphenyl)-5-methoxy-8-(3-methylbut-2-enyl)chroman-4-one	354.43	4.41	48.09	0.8	0	0.39
	MOL006613	Kushenin	286.3	2.39	47.62	0.71	0.35	0.38
	MOL006619	Kushenol J	580.59	−0.87	51.39	−2.2	−3.12	0.74
	MOL006620	Kushenol J_qt	286.3	2.27	50.86	0.24	−0.27	0.24
	MOL006622	Kushenol O	562.57	−0.56	42.41	−1.67	−2.25	0.76
	MOL006623	Kushenol T	442.55	4.46	51.28	−0.05	−0.95	0.64
	MOL006626	Leachianone G	356.4	3.89	60.97	0.33	−0.36	0.4
	MOL006627	Lehmanine	246.39	1.11	62.23	1.18	1.25	0.25
	MOL006628	(+)-Lupanine	248.41	1.42	52.71	1.16	1.19	0.24
	MOL006630	Norartocarpetin	286.25	2.07	54.93	0.14	−0.74	0.24
	MOL000456	Phaseolin	322.38	3.46	78.2	1.09	0.39	0.73
	MOL006649	Sophranol	264.41	0.67	55.42	0.6	0.68	0.28
	MOL006650	(−)-Maackiain-3-O-glucosyl-6′-O-malonate	532.49	0.7	48.69	−1.45	−2.14	0.52
	MOL006652	Trifolrhizin	462.44	0.95	48.53	−0.85	−1.65	0.74
	MOL000098	Quercetin	302.25	1.5	46.43	0.05	−0.77	0.28

Polygoni Cuspidati Rhizoma et Radix	MOL013288	Picralinal	366.45	1.8	58.01	0.23	−0.21	0.75
	MOL002259	Physcion diglucoside	608.6	−0.91	41.65	−2.64	−3.43	0.63
	MOL002268	Rhein	284.23	1.88	47.07	−0.2	−0.99	0.28
	MOL002280	Torachrysone-8-O-beta-D-(6′-oxayl)-glucoside	480.46	0.64	43.02	−1.23	−1.84	0.74
	MOL000492	(+)-Catechin	290.29	1.92	54.83	−0.03	−0.73	0.24
	MOL000098	Quercetin	302.25	1.5	46.43	0.05	−0.77	0.28

Angelicae Sinensis Radix	MOL000449	Stigmasterol	412.77	7.64	43.83	1.44	1	0.76

Galla Chinensis	MOL000569	Digallate	322.24	1.53	61.85	−0.76	−1.52	0.26

*Taraxacum mongolicum*	1	Calcium						
	2	Choline						
	3	Copper						
	4	Inulin						
	5	Iron						
	6	Manganese						
	7	Myristic acid						
	8	Palmitic acid						
	9	Potassium						
	10	Quercetin						
	11	Rutin						
	12	Stearic acid						
	13	Vitamin C						
	14	Zinc						

MW, molecular weight; *A* log *P*, water partition coefficient; OB, human oral bioavailability; Caco-2, Caco-2 permeability; BBB, blood-brain barrier; DL, drug-likeness.

**Table 2:** The number of target genes for each disease in CTD and the number of overlapped target genes.

CTD disease name	Gene count	Intersection count
Skin ulcer	16926	312
Pyoderma gangrenosum	2269	82
Acute pain	1107	67
Surgical wound dehiscence	484	28
Cutaneous fistula	423	17
Varicose ulcer	262	13
Surgical wound infection	157	10
Wound infection	149	17

**Table 3:** The functional enrichment results for the overlapped target genes.

Category	Term	Count	*P* value
KEGG	hsa04610: complement and coagulation cascades	31	1.90*E* − 28
KEGG	hsa01130: biosynthesis of antibiotics	18	1.70*E* − 04
KEGG	hsa04066: HIF-1 signaling pathway	11	5.15*E* − 04
KEGG	hsa04612: antigen processing and presentation	7	0.02474
GO BP	GO:0002576∼platelet degranulation	28	5.95*E* − 24
GO BP	GO:0006958∼complement activation, classical pathway	24	2.80*E* − 19
GO BP	GO:0030449∼regulation of complement activation	14	1.91*E* − 15
GO BP	GO:0006956∼complement activation	17	4.28*E* − 12
GO BP	GO:0045087∼innate immune response	33	1.94*E* − 11
GO BP	GO:0006957∼complement activation, alternative pathway	8	1.04*E* − 09
GO BP	GO:0007597∼blood coagulation, intrinsic pathway	8	1.79*E* − 08

## Data Availability

The data used to support the findings of this study are available from the corresponding author upon request.

## Supplementary Materials

Supplementary Materials The direct target proteins of chemical ingredients of the five herbal medicines in CYXX prescription in the DrugBank database.
